# Thoracolumbar fascia ultrasound shear strain differs between low back pain and asymptomatic individuals: expanding the evidence

**DOI:** 10.1186/s13244-024-01895-2

**Published:** 2025-01-15

**Authors:** Norio Tomita, Marie-Hélène Roy-Cardinal, Boris Chayer, Stacey Daher, Ameer Attiya, Aline Boulanger, Nathaly Gaudreault, Guy Cloutier, Nathalie J. Bureau

**Affiliations:** 1 Laboratory of Biorheology and Medical Ultrasonics, Centre hospitalier de l’Université de Montréal Research Center, Montreal, QC Canada; 2 Institute of Biomedical Engineering, Université de Montréal, Montreal, QC Canada; 3 Faculty of Medicine, Université de Montréal, Montreal, QC Canada; 4 Department of Anesthesiology, Université de Montréal, Montreal, QC Canada; 5 Pain Clinic, Centre Hospitalier de l’Université de Montréal (CHUM), Montreal, QC Canada; 6 Faculty of Medicine and Health Sciences, Université de Sherbrooke, Sherbrooke, QC Canada; 7 Centre hospitalier Universitaire de Sherbrooke Research Center, Sherbrooke, QC Canada; 8 Department of Radiology, Radio-Oncology and Nuclear Medicine, Université de Montréal, Montreal, QC Canada; 9 Department of Radiology, CHUM, Montreal, QC Canada; 10 Laboratory of Clinical Image Processing, CRCHUM, Montreal, QC Canada

**Keywords:** Fascia, Low back pain, Ultrasound, Shear strain imaging, Elastography

## Abstract

**Objectives:**

To compare thoracolumbar fascia (TLF) shear strain between individuals with and without nonspecific low back pain (NSLBP), investigate its correlation with symptoms, and assess a standardized massage technique’s impact on TLF shear strain.

**Methods:**

Participants were prospectively enrolled between February 2021 and June 2022. Pre- and post-intervention TLF ultrasound and pain/disability questionnaires were conducted. Cumulated (C|ShS|_L_) and maximum (Max|ShS|_L_) shear strain parameters were computed from radiofrequency data, and TLF thickness was measured on reconstructed B-mode images. Statistical analysis included linear mixed-effects regression.

**Results:**

Thirty-two NSLBP participants (mean age, 57 ± 9 years [standard deviation]; 21 women) and 32 controls (51 ± 10 years; 22 women) (*p* = 0.02) were enrolled. The mean shear strain was higher in NSLBP participants (C|ShS|_L_: 327.1% ± 106.0 vs 290.2% ± 99.8, *p* < 0.0001; Max|ShS|_L_: 8.1% ± 2.8 vs 7.0% ± 2.4, *p* < 0.0001) than controls, while mean TLF thickness (1.6 mm ± 1.0 vs 1.5 mm ± 0.9; *p* = 0.43) was comparable. Elastography parameters correlated with pain [C|ShS|_L_ estimate [β], 0.01 [95% CI: 0.002, 0.02]; *p* = 0.02); Max|ShS|_L_ [β]_,_ 0.003 [95% CI: 0.001, 0.005]; *p* < 0.001)] and disability [C|ShS|_L_ [β] 0.02 [95% CI: 0.005, 0.03]; *p* = 0.009); Max|ShS|_L_ [β] 0.003 [95% CI: 0.001, 0.006]; *p* = 0.002)] scores. Neither C|ShS|_L_ (β, 0.13 [−0.27, 0.53]; *p* = 0.53) nor Max|ShS|_L_ (β, −0.02 [−0.10, 0.05]; *p* = 0.59) changed post-intervention.

**Conclusion:**

Individuals with NSLBP demonstrated elevated TLF shear strain compared to controls, with similar TLF thickness. The shear strain correlated with pain and disability scores, yet a brief massage did not influence shear strain.

**Trial registration:**

Clinicaltrials.gov, NCT04716101. Registered 14 January 2021, https://clinicaltrials.gov/study/NCT04716101.

**Critical relevance statement:**

Ultrasound shows elevated TLF shear strain in lower back pain sufferers compared to controls. This correlates with symptoms, suggesting a role as a pain generator. Further investigation into its anatomy, mechanical characteristics, and pathophysiology is crucial for better understanding.

**Key Points:**

- Structural and mechanical alterations of the TLF may contribute to low back pain.
- Elevated TLF lateral shear strain was found in patients with NSLBP.
- A brief standardized massage therapy technique did not influence elastography parameters.

**Graphical Abstract:**

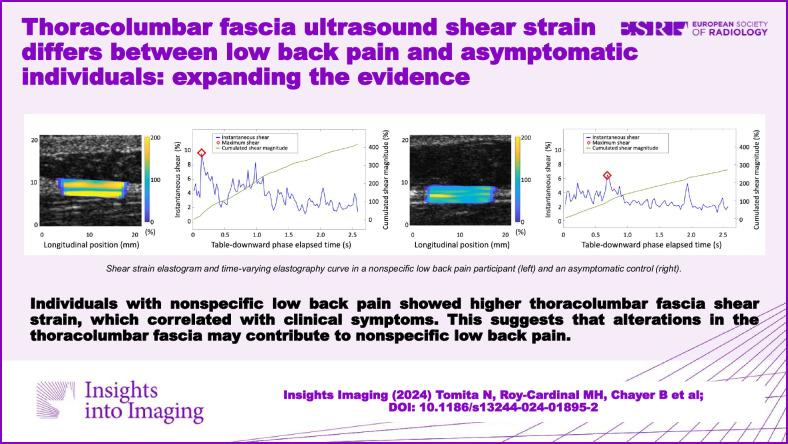

## Introduction

Low back pain refers to discomfort in the posterior side of the body between the 12th rib and the lower gluteal fold, with or without pain extending to the lower extremities [1]. It affects 7.5% of the global population, causing disability, healthcare expenditure, and reduced work productivity [2]. Unlike specific conditions such as fractures, spondylarthritis, spinal stenosis, radiculopathy, malignancy, or infection [3, 4], which can cause low back pain, 85–95% of cases seen in primary care settings are diagnosed as nonspecific low back pain (NSLBP) [5].

**Fig. 1:**
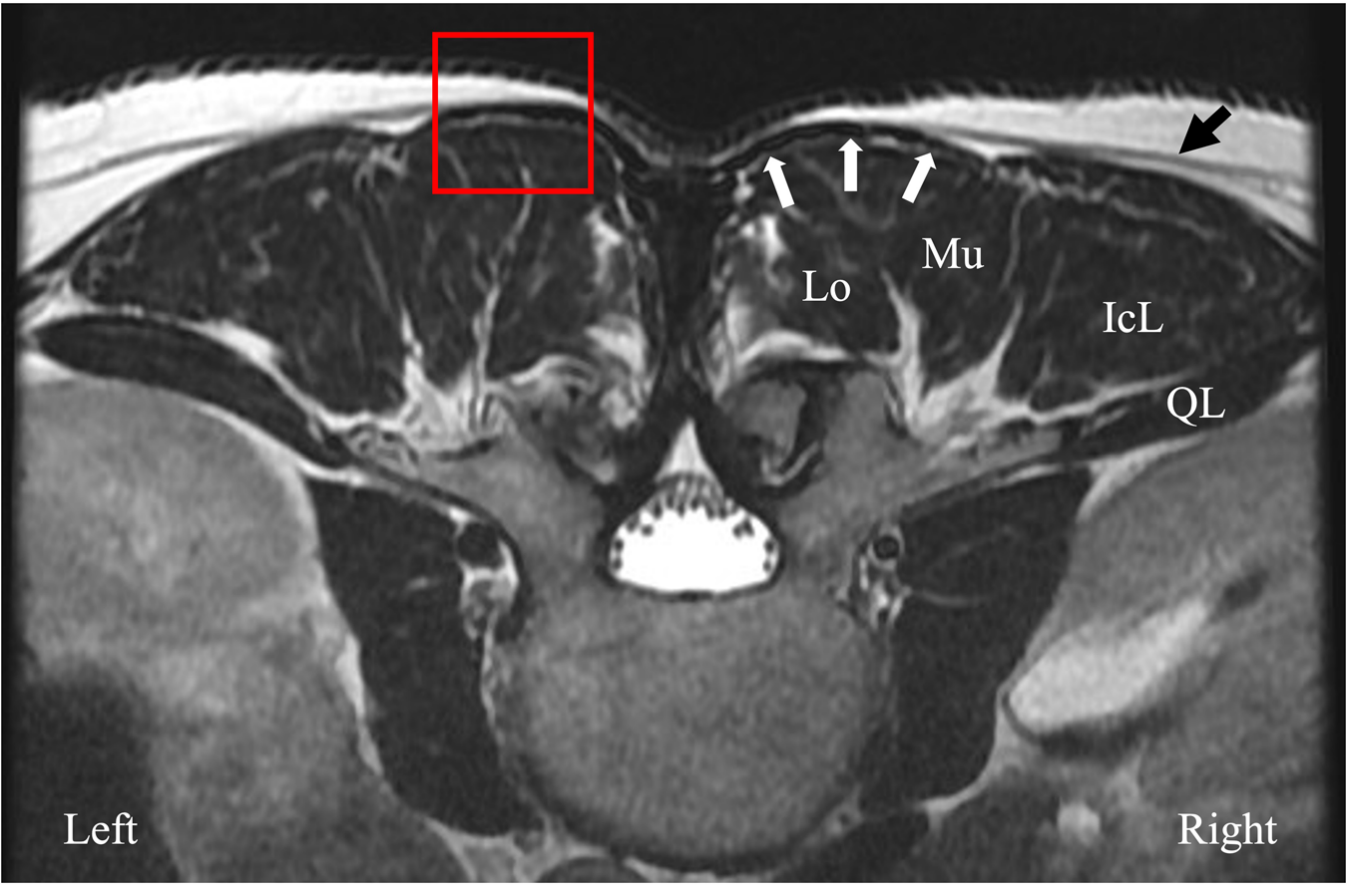
MR imaging of paraspinal soft tissues. Transverse FSE T2 MR image at the L2–L3 interspinous space in a 40-year-old man not involved in the study. The image is displayed to represent a subject in the prone position. The combined aponeuroses of the latissimus dorsi and serratus posterior inferior muscles (not shown here, located laterally) form the superficial lamina of the TLF (black arrow). The deep lamina of the TLF (not shown) is a circular fascia encapsulating the paraspinal muscles. The multifidus and longissimus muscles (ES muscles) are covered by dense ES aponeurosis, highlighted by white arrows. This aponeurosis lies deep in the TLF and attaches to the spinous processes and supraspinous ligament. The red square indicates the region at the peak point of the ES muscles examined by ultrasound. The soft tissue layers included in the square encompass the dermis, subcutaneous adipose tissue, TLF, ES aponeurosis, and muscle depicted on ultrasound in Fig. 3. QL, quadratus lumborum; IcL, iliocostalis lumborum; Mu, multifidus; Lo, longissimus; TLF, thoracolumbar fascia

The thoracolumbar fascia (TLF) is a well-organized, richly innervated fibrous tissue that interacts with muscles, providing spinal stability while transmitting forces between the upper and lower body during movement [6]. The TLF’s posterior layer comprises superficial and deep laminae. The former arises from the aponeurosis of the latissimus dorsi and serratus posterior inferior muscles, while the latter includes a fibrous retinaculum encapsulating paraspinal muscles [6]. Intervening layers of hyaluronan-rich loose connective tissue [7] facilitate movement between the TLF laminae and between the TLF and the underlying erector spinae (ES) muscles aponeurosis (Fig. 1).

Research suggests that TLF structural and mechanical alterations may contribute to NSLBP by potentially impacting mechanoreceptor input or causing irritation to nociceptive nerve endings within the TLF [8, 9]. Previous studies employed various methods, including 3D [10, 11] and 2D [12–15] B-mode ultrasound and elastography [12, 14, 15], to quantify TLF mechanics and investigate behaviors like displacement [11–13] and shear strain (ShS) [14, 15]. The effects of manual therapies on these characteristics were also explored [11–13, 15]. Notably, Langevin et al [14] found 20% lower TLF ShS in individuals with NSLBP than in controls. Vining et al [15] suggested potential gender-specific variations in ShS changes following manual therapy. However, these studies often had small sample sizes [11, 12, 15] or included only asymptomatic participants [12, 13], and much remains to be understood about the TLF’s structure, mechanical properties, and potential role as a pain generator.

This study aimed to compare TLF ultrasound ShS and the thickness of paraspinal soft tissue layers between individuals with and without NSLBP, examine the relationship between TLF ShS and symptoms, and assess the impact of a standardized massage therapy technique on TLF ShS. Our hypothesis posited lower TLF ShS in individuals with NSLBP compared to controls.

## Materials and methods

This single-center prospective study was approved by our institution’s ethics committee (CE 19.358) and was registered to Clinicaltrials.gov (NCT04716101. Registered 14 January 2021. https://clinicaltrials.gov/study/NCT04716101). Written informed consent was obtained from all participants.

### Participants

Between February 2021 and June 2022, individuals meeting inclusion criteria—adults aged 18–75 with low back or referred pain above or just below the gluteal fold (≥ 3/10 on a numerical pain rating scale) lasting at least 3 months, present at least 50% of the time during the day—were recruited by fellowship-trained anesthesiologist (A.B.) or musculoskeletal radiologist (N.J.B.) with 25 years of experience, at our institution’s Pain Clinic and Radiology Spinal Intervention Unit. Exclusion criteria included previous back or lower extremity injury, spinal surgery, pain attributable to specific known pathology, corticosteroid use or lumbar spine injection in the past 3 months, and pregnancy. Volunteers without a history of low back pain or chronic pain limiting daily activities and meeting exclusion criteria were recruited through advertisements.

A research assistant collected demographic data and measured weight, height, and lumbar spine range of motion following the Schober test method by Tousignant et al [16]. Additionally, participants completed three self-administered questionnaires: the International Physical Activity Questionnaire [17], the Brief Pain Inventory [18], and the Oswestry disability index [19].

### Ultrasound scanning

**Fig. 2:**
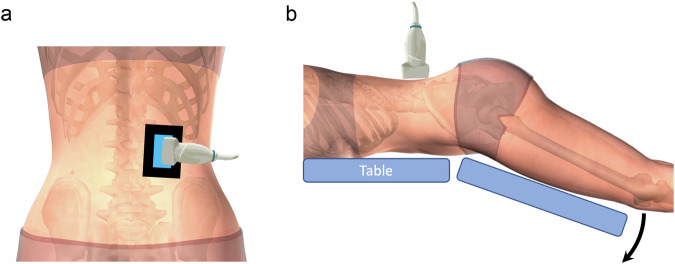
Ultrasound scanning. **a** A urethane resin plate, one cm-thick and rectangular (depicted by the black rectangle), with a customized hole to accommodate the transducer’s surface, was positioned on the paraspinal muscles at the peak point of the ES muscle, specifically at the L2–L3 interspinous level. Echo gel (blue layer) was used, and the radiologist conducted sagittal plane scans while utilizing the plate to stabilize the transducer. **b** Participants lay prone on an articulated motorized table, aligning their iliac crests with the hinge point. Scans were performed while the lower extremities underwent passive movement, as the distal portion of the table descended 20° before returning upward to the neutral position

The radiologist conducted radiofrequency (RF) data acquisitions using a system equipped with a 12L5 linear array transducer (Terason t3000 v4.7.7, Terason Ultrasound, Burlington, USA). Participants were prone on an articulated motorized table (Echo Flex Model 4800, Ibiom Instruments, Sherbrooke, Canada), aligning the iliac crests with the hinge point of the table (Fig. 2).

**Fig. 3:**
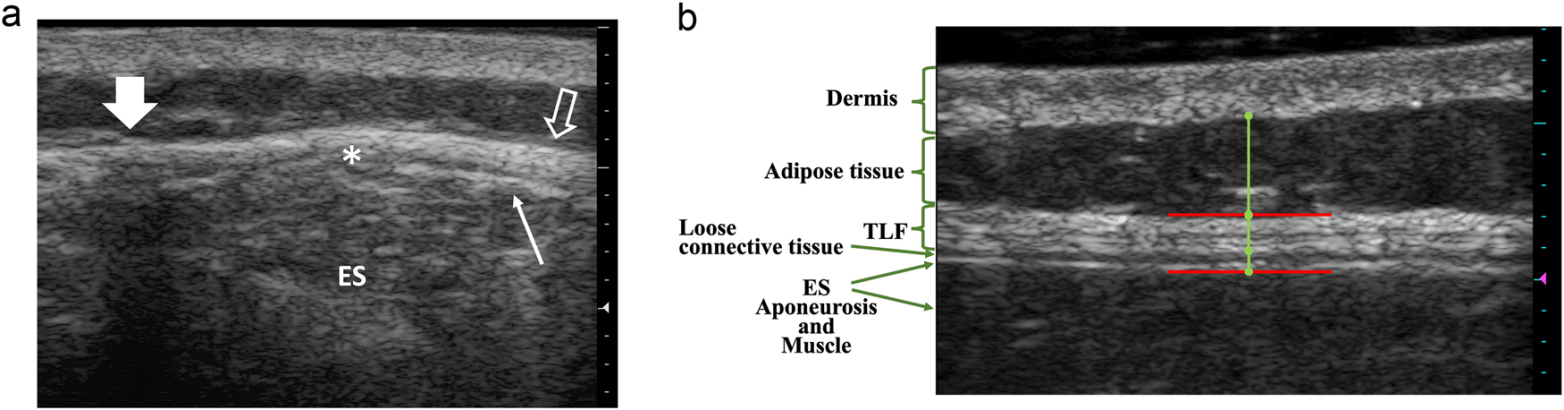
Ultrasound imaging and layer identification of paraspinal soft tissues. **a** Transverse (short-axis) and (**b**) longitudinal (long-axis) B-mode images of the right paraspinal soft tissues in a 50-year-old man with NSLBP. **a** The transverse image at the L2–L3 interspinous space (large arrow) shows the ES muscles with their hyperechoic aponeurosis (thin arrow) and the overlying TLF (open arrow). The peak convexity of the ES muscles (asterisk) was marked on the skin, and scanning was performed at this marked position in the longitudinal plane. **b** The longitudinal image displays the paraspinal tissues’ ultrasound anatomy, including the dermis, subcutaneous adipose tissue, multilaminar TLF, hypoechoic hyaluronan-rich loose connective tissue layer, hyperechoic ES aponeurosis, and muscles. For outlining paraspinal tissue layers, the medical students placed dots along the soft tissue layer margins on the image center’s first frame of the table-stationary phase. An algorithm then measured the thickness of the subcutaneous adipose tissue, TLF, and juxtamuscular zone (vertical green lines). The juxtamuscular zone included the hypoechoic hyaluronan-rich loose connective tissue and the hyperechoic ES aponeurosis. Distances were recorded in pixels and converted to millimeters using a 0.026 mm/pixel resolution. On the middle frame of the table-downward phase, two 10-mm red lines were positioned at the upper TLF and lower ES aponeurosis margins (shown together for convenience), defining the region of interest for ShS calculation. TLF, thoracolumbar fascia; NSLBP, nonspecific low back pain

At the L2–L3 interspinous level, we identified the peak point of the ES muscle on a transverse B-mode image and marked it on the skin (Fig. 3). Subsequently, we placed a one cm-thick rectangular-shaped urethane resin plate featuring a hole tailored to the transducer’s surface at the marked position. The hole was filled with echo gel. The radiologist used the plate as a stabilizer to prevent longitudinal and lateral transducer movements during scanning. The image (range 3.0–6.0 cm) and focus (range 1.3–4.5 cm) depths were adjusted to the participants’ body habitus with frequency preset “middle” and frame rate between 30.6 Hz and 48.6 Hz.

The radiologist scanned in the longitudinal plane, adjusting the transducer perpendicular to the hyperechoic ES aponeurosis. During passive movement of the lower extremities, a 10-s cine loop was captured as the distal part of the table descended 20° at an angular speed of 7°/s, followed by an upward return to the neutral position. This 10-s cine loop comprised a 4-s table-stationary phase, a 3-s table-downward phase, and a 3-s table-upward phase. Both sides of the spine were scanned three times, totaling six cine loops per participant, with each acquisition spaced one minute apart.

### Paraspinal tissue thickness measurement and RF data analysis

RF datasets were analyzed with custom MATLAB programs (vR2022a, Mathworks Inc., MA, USA). The radiologist trained two medical students (S.D., A.A.) to outline anatomical structures on B-mode reconstructed images from ten consecutive US cine loops. All were blinded to the participant group. Subsequently, the students completed the same task for the remaining datasets. On the first frame of the table-stationary phase, they measured the thickness of the subcutaneous adipose tissue, TLF, and juxtamuscular zone encompassing the loose connective tissue layer and the ES aponeurosis. Additionally, on the middle frame of the table-downward phase, they traced a 10-mm long horizontal line at the superficial margin of the TLF and the deep margin of the ES aponeurosis. The region between these two lines defined the region of interest (ROI) used for ShS calculation (Fig. 3).

The TLF’s ShS was computed using the Lagrangian Speckle Model Estimator, an ultrasound elastography analysis algorithm. This method was previously validated [20] and used to assess atheromatous plaque vulnerability [21–23], lung kinetics [24], and single-cell mechanics [25]. Unlike methods relying on B-mode images, this algorithm calculates parameters based on RF data, ensuring that ShS results are unaffected by scanner settings and image post-processing techniques, thus improving accuracy. Additionally, RF processing enhances the precision of tissue movement tracking.

The analysis involved the measurement window sliding over the ROI in the table-downward and upward phases of RF data. A cross-correlation method compensated for translating the measurement window between successive RF images. Subsequently, 2D strain components were computed using an extended version of the optical flow equation [20]. These components included axial and lateral translations, strain, and ShS, where “axial” referred to the direction along the ultrasound beam (vertical in the image), and “lateral” denoted the horizontal direction perpendicular to the axial direction in the image and the anatomical longitudinal direction [23, 26]. We extracted the time-varying instantaneous absolute lateral ShS among those parameters to characterize the TLF’s sliding movement. Here, “instantaneous” denotes measurements between successive RF frames, and the absolute value signifies the magnitude of the shear movement, irrespective of its direction.

**Fig. 4:**
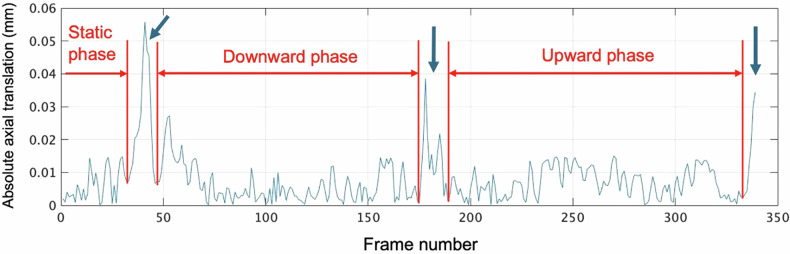
Axial translation curve over time during table downward and upward movements. This graph shows the absolute vertical displacement over time for a 74-year-old asymptomatic male volunteer, highlighting the stationary, downward, and upward table phases. Note that only a segment of the 10-s cine loop is shown. The arrows indicate that peaks in axial translation are due to table vibrations at the start of the downward movement, at the table reversal point, and at the end of the upward movement. For RF data analysis, the stable intervals during the downward and upward table movements were determined by excluding these three axial displacement peaks. Consequently, the table’s downward and upward intervals are not continuous and are analyzed separately

Vibrations at the start and end of the table’s movements caused axial translation noise. We used a time-varying axial translation curve to identify stable intervals of lateral TLF movement, excluding peak translations (Fig. 4). The resulting intervals in the table-downward phase [NSLBP 100.49 ± 13.87 vs controls 105.78 ± 16.26 (*p* = 0.27)] and the table-upward phase [NSLBP 98.45 ± 16.51 vs controls 99.94 ± 17.32 (*p* = 0.41)] had a similar number of frames. We computed the cumulative absolute lateral ShS magnitude (C|ShS|L) and the maximum absolute lateral ShS (Max|ShS|L) over these intervals.

### Intra-operator reliability

Intra-operator reliability for data acquisition and computation was evaluated using table-downward interval RF data from the pre-and post-sham intervention cases, encompassing data from 15 NSLBP and 16 control participants.

### Intervention: massage or sham technique

Participants were randomly assigned in a 1:1 ratio to receive either a standardized massage therapy or a sham technique. A certified therapist (N.T.) with 10 years of experience performed the massage technique by applying deep and slow compression and shearing forces to the participant’s lower back, moving from the iliac crest to the lower ribs, following inter-jurisdictional practice competencies guidelines [27]. This included ten strokes along the right and left ES muscles’ medial, middle, and lateral parts, totaling 30 strokes per side. A custom-made force sensing system comprising a unidirectional reusable flexible force sensor (FlexiForce Standard Model A301, Tekscan, MA, USA) affixed to a massage tool (Deep Pressure Thumb Saver Massager, MIGLEO, Shenzhen, China) was used to measure and calibrate the force during the massage strokes. Connected to a computer through an amplifier (non-inverting op-amp circuit) and an analog-to-digital converter (NIUSB-6251, National Instruments Corp. TX, USA), the force sensor gauged the therapist’s thumb’s force on the massage tool in Newtons using a pre-calibrated force-voltage relationship. Before the massage, the therapist determined the pressure threshold causing discomfort. During the massage, the therapist exerted pressure between the upper discomfort threshold and two-thirds of that value to ensure participant comfort. We standardized the force applied to relative intensity, mirroring clinical practice. The therapist performed the sham technique by placing one hand on the ES muscles without exerting pressure sequentially on the right and left ES muscles. Both interventions were conducted over a hospital gown, each lasting 2.5 min. Ultrasound data acquisitions were repeated immediately after the intervention.

### Sample size and statistical analyses

The study included a convenient sample of 60 participants equally divided into two groups, with 32 participants per group to account for potential failed examinations. Descriptive statistics were used to analyze demographic and clinical characteristics, ShS parameters, and the thickness of the paraspinal soft tissues. The Shapiro–Wilk test assessed data distribution, and square root transformations were utilized for non-normally distributed data. Linear mixed-effects models were employed to analyze between-group comparisons of ShS parameters, examine the impact of the interventions on ShS parameters, and investigate the correlation between ShS parameters and pain and disability scores. Intra-operator reliability was assessed using the intraclass coefficient correlation (ICC) with 95% confidence intervals. One author (MHRC) conducted the statistical analyses using R software (v4.2.1, The R Foundation for Statistical Computing, Vienna, Austria) with a two-tailed test and a significance level of 0.05.

## Results

**Fig. 5:**
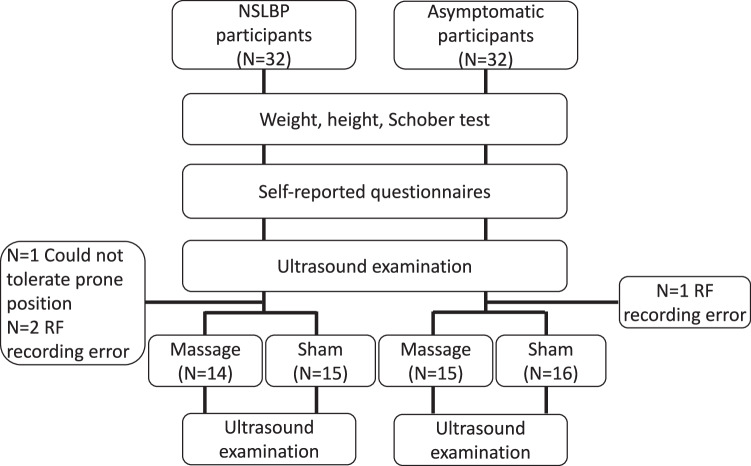
The study flow diagram. The Schober test for assessing the lumbar spine range of motion was conducted following the method by Tousignant et al [16]. The self-administered questionnaires included the International Physical Activity Questionnaire [17], the Brief Pain Inventory [18], and the Oswestry disability index [19]. NSLBP, nonspecific low back pain

Figure 5 presents the study’s flow diagram.

### Participants characteristics

Thirty-two NSLBP participants (mean age, 57 ± 9 years, 21 women) and 32 controls (51 ± 10 years, 22 women) (*p* = 0.02) were enrolled. The NSLBP group exhibited higher BMI (*p* = 0.03) and reduced lumbar spine range of motion (*p* = 0.003) compared to controls. NSLBP participants reported greater pain and disability (Brief Pain Inventory: *p* < 0.001; Oswestry Disability Index: *p* < 0.001), while both groups showed similar activity levels (International Physical Activity Questionnaire: *p* = 0.24) (Table 1).

**Table 1:** Demographic and clinical characteristics of the participants

		Groups		
Variables		NSLBP	Controls	*p* value*
*N*		32	32	
Sex (%)	Men	11 (34.4)	10 (31.2)	1.00
	Women	21 (65.6)	22 (68.8)	
Age, years (mean ± SD, range)		57.3 ± 9.3, 40–73	51.4 ± 9.9, 38–74	0.02
Height, cm (mean ± SD, range)		165.2 ± 8.6, 147–178	163.7 ± 7.0, 148–178	0.43
Weight, kg (mean ± SD, range)		85.3 ± 19.9, 51.3–138.0	74.3 ± 16.4, 49.0–127.0	0.02
BMI, kg/m^2^ (mean ± SD, range)		31.4 ± 7.1, 22.3–47.9	27.8 ± 6.0, 17.9–49.6	0.03
Brief pain inventory, (mean ± SD, range)		52.4 ± 23.4, 9–109	2.0 ± 2.7, 1–13	< 0.001
Oswestry disability index, % (mean ± SD, range)		35.3 ± 16.2, 10–78	0.7 ± 2.0, 0–8	< 0.001
International physical activity questionnaire (%)	High (inactive)	6 (19.4.0)	12 (37.5)	0.24
	Moderate (minimally active)	10 (32.3)	11 (34.4)	
	Low (highly active)	14 (45.2)	9 (28.1)	
Schober test, cm (mean ± SD, range)		4.7 ± 1.3, 2.0–6.5	5.7 ± 1.4, 3.5–8.0	0.003

*N* number, *SD* standard deviation, *BMI* body mass index, *NSLBP* nonspecific low back pain participants

^*^ Fisher’s exact or Student *t*-test. A higher score on the Brief Pain Inventory questionnaire and the Oswestry Disability Index indicates higher levels of pain and functional disability, respectively. The International Physical Activity Questionnaire evaluates physical activity levels. A lower score on the Schober test indicates a lower lumbar spine range of motion

Four participants were excluded from the analysis: one NSLBP participant could not tolerate a prone position on the examination table, and two NSLBP participants and one control had RF recording errors—consequently, the analysis comprised data from 29 NSLBP participants and 31 controls.

### Intra-operator reliability

The measurements’ reliability was excellent, with ICC values of 0.95 (95% CI: 0.90, 0.98) for C|ShS|_L_ and 0.91 (95% CI: 0.81, 0.96) for Max|ShS|_L._

### Paraspinal soft tissue thickness

The NSLBP group exhibited thicker subcutaneous adipose tissue (mean 13.3 ± 9.0 mm; range: 1.2–44.4 vs 9.3 ± 5.3 mm; 1.9–23.0; *p* < 0.001) and a thicker juxtamuscular zone (1.2 ± 0.6 mm; 0.4–5.1 vs 1.1 ± 0.3 mm; 0.3–1.8; *p* = 0.02). However, TLF thickness (1.6 ± 1.0 mm; 0.5–5.0 vs 1.5 ± 0.9 mm; 0.4–5.2; *p* = 0.43) was comparable between the groups.

### ShS elastography

There was no difference in C|ShS|L and Max|ShS|L values between the table’s downward and upward phases in both groups (Table 2). Since the table-upward phase results do not affect the study’s outcomes, only the table-downward phase results are presented.

**Table 2:** Comparison of ShS elastography parameters in downward and upward phases

Group	Variables	Downward phase	Upward phase	*p* value
NSLBP	C|ShS|_L_ (%) mean ± SD, range	327.1 ± 106.0 [128.9–660.1]	324.3 ± 109.7 [124.2–737.5]	0.54
	Max|ShS|_L_ (%) mean ± SD, range	8.1 ± 2.8 [3.4–20.0]	8.0 ± 3.0 [3.0–23.7]	0.60
Controls	C|ShS|_L_ (%) mean ± SD, range	290.2 ± 99.8 [105.6–581.3]	283.7 ± 110.7 [90.2–845.0]	0.24
	Max|ShS|_L_ (%) mean ± SD, range	7.0 ± 2.4 [2.1–15.6]	7.0 ± 2.4 [1.9–16.6]	0.94

*SD* standard deviation, *NSLBP* nonspecific low back pain participants, *C|ShS|*_*L*_ cumulated absolute lateral shear strain magnitude, *Max|ShS|*_*L*_ maximum absolute lateral shear strain

**Fig. 6:**
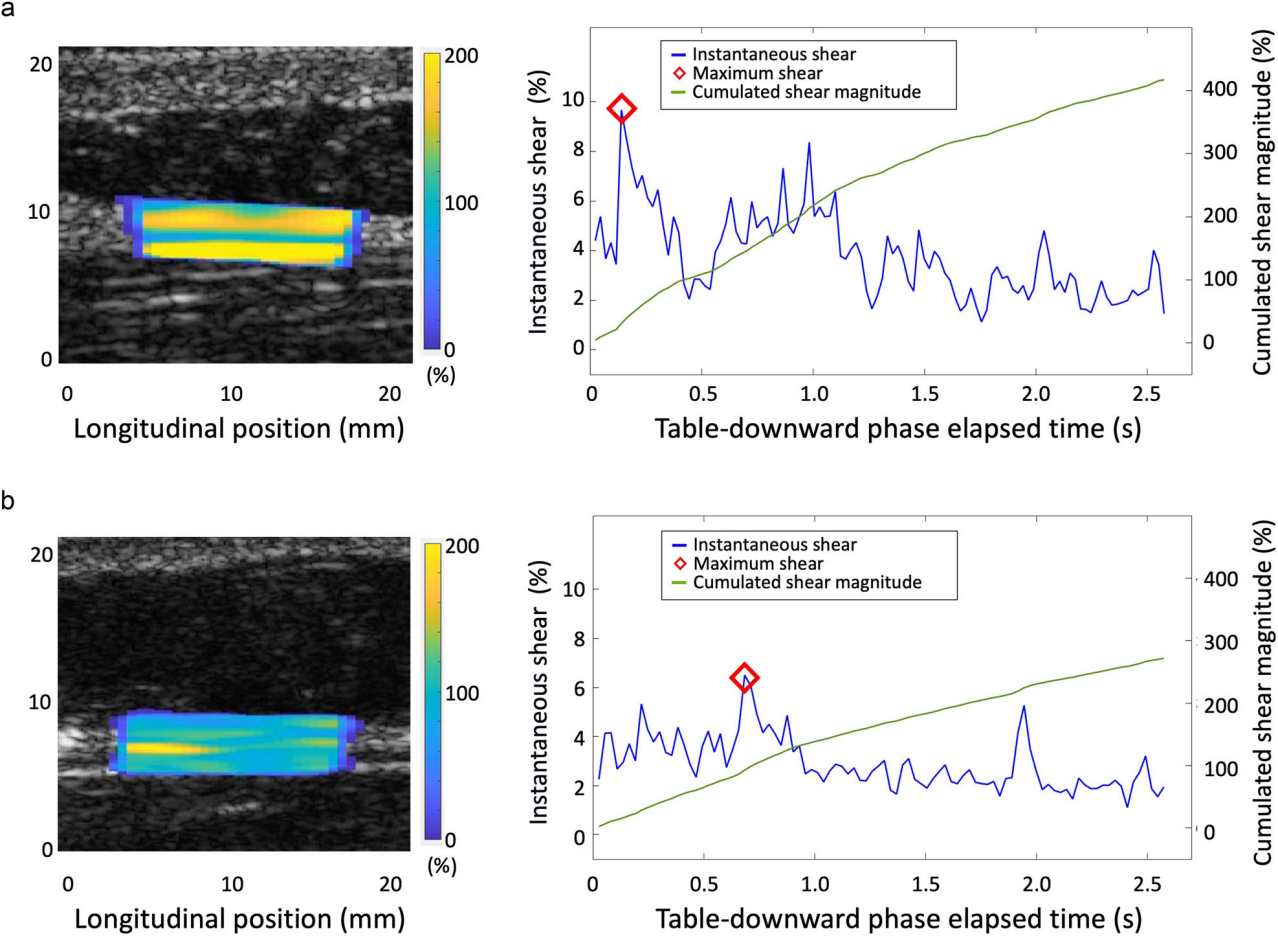
Table-downward phase ShS elastograms and time-varying elastography curves. **a** depicts the ShS elastogram and time-varying elastography curves from a 59-year-old woman with NSLBP, while **b** shows data from a 38-year-old asymptomatic woman volunteer. The shear strain elastograms display the C|ShS|_L_ within the ROI, superimposed on the reconstructed 2D B-mode images, with higher values noted in the NSLBP participant. The blue curves represent the instantaneous absolute lateral ShS averaged within the ROI of each frame, with the red marker indicating the Max|ShS|L% recorded during the table-downward interval. The green curves represent the magnitude of cumulative instantaneous absolute lateral shear strain (C|ShS|L%). Max|ShS|L% and C|ShS|L% parameters are higher in the NSLBP participant. NSLBP, nonspecific low back pain; ROI, region of interest

The NSLBP group exhibited significantly higher C|ShS|_L_ (mean 327.1 ± 106.0% vs 290.2 ± 99.8%, *p* < 0.001) and Max|ShS|_L_ (8.1 ± 2.8% vs 7.0 ± 2.4%, *p* < 0.001) values compared to controls (Fig. 6 and media files). In the regression model, while controlling for Age, BMI, and Frame rate, Group and Sex emerged as the most influential predictors on the elastography parameters, indicating elevated values among NSLBP participants and women compared to controls and men, respectively (Table 3). To further investigate whether BMI influenced the effect of Group on the ShS parameters, we tested a model that included an interaction term (Group × BMI). This model aimed to explore the potential moderation effect of BMI on the observed relationships. However, the analysis revealed no significant differences in the effect of BMI on C|ShS|_L_ (*p* = 0.06) and Max|ShS|_L_ (*p* = 0.14) between groups (Supplementary Table 1).

**Table 3:** Comparison of ShS elastography parameters between groups during the table-downward phase

Dependent variables	Predictors	Estimates	SD	*p* value	95% CI
C|ShS|_L_ (%)	Group (controls)	1.27^*a^	5.68	< 0.001	[0.69, 1.86]
	Sex (men)	1.80^*b^	5.84	< 0.001	[1.21, 2.40]
	Age	−0.04	0.29	0.006	[−0.07, −0.01]
	BMI	0.06	0.45	0.01	[0.01, 0.11]
	Frame rate	−0.04	0.66	0.21	[−0.11, 0.02]
Max|ShS|_L_ (%)	Group (controls)	0.19^*a^	0.88	< 0.001	[0.10, 0.28]
	Sex (men)	0.17^*b^	0.91	< 0.001	[0.08, 0.27]
	Age	−0.01	0.04	0.009	[−0.01, −0.001]
	BMI	0.01	0.07	0.009	[0.003, 0.02]
	Frame rate	−0.02	0.10	< 0.001	[−0.04, −0.02]

For the categorical predictor's group and sex, the reference category is indicated in parentheses

*C|ShS|L* cumulated absolute lateral shear strain magnitude, *Max|ShS|L* maximum absolute lateral shear strain, *SD* standard deviation, *95% CI* 95% confidence interval, *BMI* body mass index

^*^^a^ Positive estimate values indicate that the NSLBP group had significantly greater ShS than the control group

^*^^b^ Positive estimate values indicate that women had significantly greater ShS than men

The control group tolerated a greater massage force (mean 44.9 ± 8.6 Newtons) than NSLBP participants (32.9 ± 14.3 N) (*p* = 0.01). However, following adjustments for various variables, neither C|ShS|_L_ (*p* = 0.53) nor Max|ShS|_L_ (*p* = 0.59) changed following either intervention (Table 4). The elastography parameter values are listed in the supplementary Table 2.

**Table 4:** Effect of standardized massage therapy and sham techniques on the thoracolumbar ShS elastography parameters during the table-downward phase

Dependent variables	Predictors	Estimates	SD	*p* value	95% CI
C|ShS|_L_ (%)	Timepoint (before intervention)	0.13	5.51	0.53	[−0.27, 0.53]
	Group^*a^ (controls)	1.01	9.16	0.003	[0.35, 1.68]
	Intervention^*b^ (Sham technique)	2.18	14.05	< 0.001	[1.15, 3.20]
	Frame rate	−0.06	0.69	0.02	[−0.11, −0.01]
	Massage force	−0.05	0.35	< 0.001	[−0.08, −0.03]
	Group^*^ intervention^*c^ (controls^*^Sham technique)	0.46	11.97	< 0.001	[−0.41, 1.34]
Max|ShS|_L_ (%)	Timepoint (before intervention)	−0.02	1.06	0.59	[−0.10, 0.05]
	Group^*a^ (controls)	0.21	1.45	0.001	[0.09, 0.31]
	Intervention^*b^ (Sham technique)	0.18	2.21	0.03	[0.02, 0.34]
	Frame rate	−0.03	0.11	< 0.001	[−0.04, −0.02]
	Massage force	−0.002	0.06	0.34	[−0.01, 0.002]
	Group^*^ intervention^c^ (controls^*^ Sham technique)	0.11	1.89	0.13	[−0.03, 0.24]

The reference category is indicated in parentheses for the categorical predictor's timepoint, group, intervention, and group^*^intervention. Neither intervention had a significant effect on the thoracolumbar ShS parameters

*C|ShS|L* cumulated absolute lateral ShS magnitude, *Max|ShS|L* maximum absolute lateral shear strain, *SD* standard deviation, *95% CI* 95% confidence interval

^*a^ Positive estimate values indicate that the NSLBP group had significantly greater shear strain than the control group

^*b^ Positive estimate values indicate that the massage technique led to greater shear strain compared to the sham technique

^*c^ Positive estimate values indicate that the NSLBP group receiving the massage technique showed the greater ShS

We observed significant positive correlations between the elastography parameters and the Brief Pain Inventory questionnaire [C|ShS|_L_ (*p* = 0.02); Max|ShS|_L_ (*p* < 0.001)] and Oswestry disability index [C|ShS|_L_ (*p* = 0.009); Max|ShS|_L_ (*p* = 0.002)] scores (Table 5).

**Table 5:** Correlation between thoracolumbar ShS elastography parameters and pain and disability scores during the table-downward phase

Dependent variables	Predictors	Estimates	SD	*p* value	95% CI
C|ShS|_L_ (%)	BPI	0.01	0.10	0.02	[0.002, 0.02]
	Sex^*a^ (men)	1.85	5.92	< 0.001	[1.25, 2.46]
	BMI	0.07	0.45	0.002	[0.03, 0.12]
	Age	−0.03	0.28	0.02	[−0.06, −0.01]
Max|ShS|_L_ (%)	BPI	0.003	0.02	< 0.001	[0.001, 0.005]
	Sex^*a^ (men)	0.21	0.94	< 0.001	[0.11, 0.31]
	BMI	0.01	0.07	< 0.001	[0.008, 0.02]
	Age	−0.01	0.04	0.004	[−0.01, −0.002]
C|ShS|_L_ (%)	ODI	0.02	0.14	0.009	[0.005, 0.03]
	Sex^*a^ (men)	1.78	5.90	< 0.001	[1.18, 2.40]
	BMI	0.07	0.45	< 0.001	[0.02, 0.12]
	Age	−0.03	0.28	0.003	[−0.06, −0.003]
Max|ShS|_L_ (%)	ODI	0.003	0.02	0.002	[0.001, 0.006]
	Sex^*a^ (Men)	0.19	0.94	< 0.001	[0.10, 0.29]
	BMI	0.02	0.07	< 0.001	[0.01, 0.02]
	Age	−0.01	0.04	< 0.001	[−0.01, −0.002]

For the categorical predictor Sex, the reference category is indicated in parentheses. While controlling for Sex, BMI, and Age, the Brief Pain Inventory questionnaire (BPI) and the Oswestry Disability Index scores emerged as significant predictors of the thoracolumbar ShS parameters

*C|ShS|L* cumulated absolute lateral shear strain magnitude, *Max|ShS|L* maximum absolute lateral shear strain, *SD* standard deviation, *95% CI* 95% confident interval, *BMI* body mass index

^*a^ Positive estimate values indicate that women had significantly greater ShS than men

## Discussion

We conducted a study on TLF lateral ShS employing ultrasound elastography. Our method showed excellent intra-operator reliability. Contrary to expectations and previous findings by Langevin et al [14], our study revealed elevated ShS in participants with NSLBP compared to controls.

However, the two studies used different concepts to characterize TLF deformation: displacement and ShS. Displacement indicates how far and in what direction a point in the tissue has moved, while ShS measures angular deformation and shape change due to shearing forces. Langevin’s method calculated the normalized maximum displacement difference between two ROIs along a single plane at the TLF-ES aponeurosis interface. In contrast, our method calculated ShS by tracking the movement of all pixels within the ROI, capturing changes in shape and the relative motion of layers within the TLF and ES aponeurosis. This approach likely captured more intricate TLF mechanical behaviors. In our computation, a value approaching zero indicates uniform translation in a consistent direction, while greater absolute values suggest nonuniform translations in diverse directions.

Recent studies underscore the complex interplay between fascia and muscles for optimal myofascial unit functioning [28]. Any structural alterations or hindrances in fascial layer interactions could affect sensory signals transmitted by muscle spindles to the central nervous system and disrupt coordinated movements [29]. Brandl et al [30] investigated the association between TLF deformation, indicated by muscle-fascia junction displacement, and ES muscle contractions assessed through surface electromyography during active trunk extension. Subjects with NSLBP exhibited erratic TLF mechanical behavior, resulting in inconsistent muscle contractions, in contrast to the more regular changes observed in controls. Therefore, our identification of higher TLF ShS in NSLBP participants might stem from shifts in TLF tension and dyskinetic sliding patterns.

We showed higher ShS in women than men, aligning with previous studies [14, 15]. Contributing factors may include differences in connective tissue composition, fat distribution, hormonal influences, muscle tone, or distinct movement patterns. Elevated ShS was observed in participants with higher BMI, while lower strain was noted in older participants. These observations might be linked to variations in fat distribution, the pre-loading effect of tissues in cases of increased BMI, and age-related alterations in myofascial tissues affecting ShS dynamics.

The literature suggests that a standardized massage stroke technique may alter pressure dynamics within the hyaluronan layer, thereby potentially improving tissue sliding efficacy [31]. Griefahn et al [13] supported this concept, demonstrating the impact of active foam roller exercises on back muscles, significantly enhancing TLF sliding mobility in healthy individuals. In our study, a standardized massage technique applied maximal force within the control group. Still, no ShS changes were observed, suggesting that the technique’s brevity or limited force might not have been sufficient to trigger discernible TLF ShS changes.

We observed thicker subcutaneous adipose tissue in the NSLBP group than controls, consistent with the group’s higher BMI. The TLF thickness was similar between the NSLBP group (mean 1.6 ± 1.0 mm) and the control group (1.6 ± 0.8 mm) and aligned with the findings of Creze et al [32] in cadaveric specimens (0.96 ± 0.15 mm). Our results aligned with Wilke et al [33] regarding the juxtamuscular zone thickness, including the hyaluronan-rich loose connective tissue layer and the ES aponeurosis. Our observations suggest a thicker juxtamuscular zone in individuals with NSLBP than controls. Stecco et al [34] proposed that deep fascia thickening in patients with chronic neck pain could be due to changes in hyaluronan density and fascial viscosity within the interleaved loose connective tissue. Similarly, our findings may indicate analogous modifications in the hyaluronan-rich loose connective tissue layer between the TLF and the underlying ES aponeurosis.

Finally, we found a positive association between elastography parameters and symptoms. These findings suggest that alterations in TLF ShS might reflect underlying pathological processes or myofascial dysfunction, contributing to the experience of pain and functional impairments in individuals with NSLBP. This correlation underscores the importance of considering the TLF mechanical properties in understanding and managing NSLBP.

Our study had several limitations. The term “NSLBP” can be vague, given the multifactorial nature of its underlying causes. Although participants were recruited by physicians and previous assessments excluded identifiable pathologic causes, there remains a possibility of undiagnosed specific contributors to the pain. Our participant cohort received hospital care, which might represent a subset of individuals with more severe NSLBP despite their activity level being comparable to the volunteer group. This potential selection bias could limit the generalizability of our findings to a broader spectrum of NSLBP patients. Our sample size was moderate, potentially affecting the robustness of our results. Nevertheless, our outcomes offer preliminary quantitative data that can guide future studies in determining the appropriate sample size based on relevant endpoints.

The study found higher TLF ShS among individuals with NSLBP than controls, between-group connective tissue differences, and positive correlations between elastography parameters and symptoms, indicating a potential link between TLF alterations and NSLBP. However, a brief standardized massage technique did not immediately alter TLF ShS.

## Supplementary information

ELECTRONIC SUPPLEMENTARY MATERIAL

Media file 1

Media file 2

Legends for Media Files 1 and 2

## Data Availability

Upon request, the corresponding author can provide access to datasets analyzed in this study.

## Abbreviations

C|ShS|_L_: Cumulated absolute lateral shear strain magnitude
ES: Erector spinae
ICC: Intraclass coefficient correlation
Max|ShS|_L_: Maximum absolute lateral shear strain
NSLBP: Nonspecific low back pain
RF: Radiofrequency
ROI: Region of interest
SD: Standard deviation
ShS: Shear strain
TLF: Thoracolumbar fascia
