# Supplementary material for: Thoracolumbar fascia ultrasound shear strain differs between low back pain and asymptomatic individuals: expanding the evidence
Source: Insights Imaging. 2025 Jan 15;16:18. doi: 10.1186/s13244-024-01895-2 (PMC11735703; doi:10.1186/s13244-024-01895-2)
Supplement: Supplementary file 1 — ELECTRONIC SUPPLEMENTARY MATERIAL [file 13244_2024_1895_MOESM1_ESM.pdf]

Thoracolumbar Fascia Ultrasound Shear Strain Differs Between Low Back Pain and Asymptomatic Individuals: Expanding the Evidence

ELECTRONIC SUPPLEMENTARY MATERIAL

Supplementary Table 1. Comparison of shear strain elastography parameters between groups during the table-downward phase. The regression model includes the interaction term (Group * BMI).

**Shear strain ~ Group*BMI_centered + Sex + Age + FrameRate + (1| Sample)**						
**Dependent Variables**	**Predictors**	**Estimates**	**SD**	**p value**	**95% CI**	**VIF**
$C\|ShS\|_L$ (%)	Group (Controls)	1.28	5.65	<0.001[*c]	[0.62 1.89]	1.12
	Sex (Men)	1.82	5.82	<0.001	[1.24 2.41]	2.38
	Age	-0.04	0.29	0.01	[-0.07 -0.01]	1.06
	BMI_centered	0.01	0.65	0.69	[-0.05 0.08]	1.15
	Frame rate	-0.05	0.67	0.15	[-0.12 0.02]	1.17
	Group*BMI_centered (Controls)	0.09	0.86	0.06[*a]	[-0.01 0.17]	2.16
$Max\|ShS\|_L$ (%)	Group (Controls)	0.19	0.88	<0.001[*c]	[0.09 0.28]	1.12
	Sex (Men)	0.18	0.91	<0.001	[0.07 0.29]	2.38
	Age	-0.01	0.04	0.01	[-0.01 -0.002]	1.06
	BMI_centered	0.004	0.10	0.43	[-0.01 0.01]	1.15
	Frame rate	-0.03	0.10	<0.001	[-0.04 -0.02]	1.17
	Group*BMI_centered (Controls)	0.01	0.13	0.14[*a]	[-0.003 0.03]	2.16

For the categorical predictors Group and Sex, the reference category is indicated in parentheses. ***a**, The interaction term Group*BMI had no significant effect on the shear strain parameters; In this model, centering the BMI was used to resolve multicollinearity issues; $C|ShS|_L$ = cumulated absolute lateral shear strain magnitude; $Max|ShS|_L$ = maximum absolute lateral shear strain; SD = standard deviation; 95% CI = 95% confidence interval; BMI = body mass index; VIF = Variance Inflation Factor.

Supplementary Table 2. Shear strain elastography parameter values before and after the standardized massage therapy and sham techniques during the table-downward phase.

Dependent Variables	Time Points	Groups			
		NSLBP		Controls	
		Massage (N=14)	Sham (N=15)	Massage (N=15)	Sham (N=16)
C|ShS|L, (%) (mean±SD, range)	Before	334.6 ± 123.9<br>128.9 - 660.1	320.0 ± 86.3<br>155.0 - 523.6	282.2 ± 99.4<br>105.6 - 581.0	297.7 ± 100.1<br>109.8 - 581.3
	After	321.7 ± 105.4<br>117.2 - 580.3	316.4 ± 90.7<br>163.7 - 586.7	274.5 ± 88.2<br>126.7 - 587.5	295.5 ± 90.2<br>143.7 - 708.5
Max|ShS|L, (%) (mean±SD, range)	Before	8.2 ± 3.4<br>3.9 - 20.0	7.9 ± 2.2<br>3.4 - 13.7	6.9 ± 2.4<br>2.1 - 14.3	7.2 ± 2.4<br>2.4 - 15.6
	After	8.0 ± 3.0<br>2.9 - 17.8	8.1 ± 2.5<br>4.0 - 15.6	7.4 ± 2.7<br>2.8 - 15.4	7.0 ± 2.0<br>3.1 - 14.3

Time points = before versus after the intervention; N = number; SD = standard deviation; NSLBP = nonspecific low back pain participants; $C|ShS|_L$ = cumulated absolute lateral shear strain magnitude; $Max|ShS|_L$ = maximum absolute lateral shear strain.
