# Supplementary material for: Thoracolumbar fascia ultrasound shear strain differs between low back pain and asymptomatic individuals: expanding the evidence
Source: Insights Imaging. 2025 Jan 15;16:18. doi: 10.1186/s13244-024-01895-2 (PMC11735703; doi:10.1186/s13244-024-01895-2)
Supplement: Supplementary file 4 — Legends for Media Files 1 and 2 [file 13244_2024_1895_MOESM4_ESM.docx]

10.1186/s13244-024-01895-2 / INSI-D-24-00956R2 - Post Proof Query

Media file 1: A video illustrating thoracolumbar fascia (TLF) sliding movement in a 59-year-old woman with nonspecific low back pain.

The probe was positioned longitudinally at the peak point of the erector spinae muscle at the L2-3 interspinous level. The video, featuring the same participant in Figure 5a, captures TLF sliding movement during the table-downward and upward phases.

Media file 2: A video illustrating thoracolumbar fascia (TLF) sliding movement in a 38-year-old asymptomatic woman volunteer.

The probe was positioned longitudinally at the peak point of the erector spinae muscle at the L2-3 interspinous level. The video, featuring the same participant in Figure 5b, captures TLF sliding movement during the table-downward and upward phases.
